# Sanitation in the Field

**Published:** 1905-02-18

**Authors:** 


### Sanitation in the Field.

Among the many surprising features of the war raging in Manchuria few are likely to be more surprising to the general public than the results which have been attained by the simple expedient of giving a free hand to the officers of the Medical Department of the Japanese Army, and by supporting, instead of thwarting, them in their efforts to preserve the health of the troops committed to their care. In every previous campaign recorded in history, unless perhaps in the so-called "doctors' war" waged by the English in Abyssinia, the deaths and disablements produced by disease have far outnumbered those produced by the weapons of the enemy; and, so recently as in South Africa, our own death record from enteric fever was appalling. Our medical officers, of course, have always contended that the diseases chiefly fatal in the field might in great measure be prevented, but their voices have for the most part failed to compel the attention of the public. Pall Mall has pronounced their most reasonable requirements to be "impossible" of fulfilment, and the stupid arrogance of incompetent generals appointed to commands by the influence of "society" has decided that measures for the prevention of disease would be at once troublesome and inoperative. One man alone, Dr. Leigh Canney, has spoken on the subject of enteric fever with sufficient energy to command some portion of attention from the country, and to compel the War Office to institute some so-called experiments of such a character that they were foredoomed to failure, and were apparently only intended to furnish excuses for inaction. The inoculation of anti-typhoid serum, as proposed by Professor Wright and advocated by a succession of scientific Committees of Inquiry, has been almost forced upon the authorities, and they have responded by ordering trials of the method to be made in such a manner, and under such conditions, that Professor Wright himself has withdrawn from all participation in them, and from all responsibility for the results. While this has been our own history, the whole of the problems connected with camp sanitation appear to have been solved by the Japanese; and the health of their soldiers has been as well preserved in the field as it would have been in barracks. It has been recorded that only forty deaths from disease had occurred in General Oku's division between April and December; and Major Seaman, of the United States Volunteers, has furnished a report giving some idea of the methods by which such immunity has been obtained. He says that the prevention of disease by the careful supervision of the smallest details of subsistence, clothing, and shelter has been the first and most important duty of the Japanese Army surgeons. Nothing was so small as to escape their vigilance, or so tedious as to weary their patience. The medical officer was with the first screen of scouts, with microscope and chemicals, testing and labelling wells, so that the army which followed should drink no contaminated water. When they reached a town, he immediately made a thorough examination of its sanitary conditions, and secured the isolation of any contagious or infectious disease which might be present. A medical officer accompanied every foraging party, and, with the commissariat officer, examined the food, fruit, or vegetables offered for sale by the natives. If the food were tainted, or the fruit over-ripe, or if the water required boiling, proper notices were posted in suitable places, and the discipline was such that obedience to the orders of the medical officer was absolute. He also supervised the personal hygiene of the camp, taught the men how to cook and bathe, how to cleanse the finger nails so as to free them from bacteria, and how to live a healthy and vigorous life. It was part of the disciplinary routine to carry out his instructions in every particular, and the effect has been a practical immunity from the dysentery and fevers which customarily follow the use of improper food and the neglect of sanitation. During six months of severe fighting and exposure in a foreign country, there was only a fraction of one per cent. of loss from preventible disease. It is manifest that the contentions of military sanitarians have been completely justified, and justified without any impairment of military secrecy, promptitude, or efficiency. The ignorant "Combatant" martinet must for the future be taught to subordinate his ignorance to knowledge which has been so conspicuously successful; and the cry of Augustus to Varus must be repeated by the English people if any future military expedition should be permitted to suffer from disease after the manner that has been customary in the past.

